# The Autonomous House: A Bio-Hydrogen Based Energy Self-Sufficient Approach

**DOI:** 10.3390/ijerph6041515

**Published:** 2009-04-21

**Authors:** Shang-Yuan Chen, Chen-Yeon Chu, Ming-jen Cheng, Chiu-Yue Lin

**Affiliations:** 1 Department of Architecture, Feng Chia University, No. 100 Wenhwa Rd., Seatwen, Taichung, Taiwan 40724, R.O.C; 2 Research Center for Energy & Resources, Feng Chia University; E-Mail: cychu@fcu.edu.tw; 3 Department of Architecture, Feng Chia University; E-Mail: mjcheng@fcu.edu.tw; 4 Department of Environmental Engineering & Science, Feng Chia University; E-Mail: cylin@fcu.edu.tw

**Keywords:** Hydrogen production by dark fermentation, proton exchange membrane fuel cells, passive design, active equipment, green energy technology

## Abstract

In the wake of the greenhouse effect and global energy crisis, finding sources of clean, alternative energy and developing everyday life applications have become urgent tasks. This study proposes the development of an “autonomous house” emphasizing the use of modern green energy technology to reduce environmental load, achieve energy autonomy and use energy intelligently in order to create a sustainable, comfortable living environment. The houses’ two attributes are: (1) a self-sufficient energy cycle and (2) autonomous energy control to maintain environmental comfort. The autonomous house thus combines energy-conserving, carbon emission-reducing passive design with active elements needed to maintain a comfortable environment.

## 1. Background and Goals

Efforts to promote the application and utility of energy since the beginning of the 20th century have resulted in the invention of many types of energy-consuming active equipment and household appliances. Due to growing awareness of the vulnerability of the Earth’s ecological environment, environmentalists have promoted energy-conserving passive design since the late 20th century. In the wake of the greenhouse effect and global energy crisis, the dawn of the 21st century has forced the world to confront the contradiction between energy-conserving sustainable passive design and energy-consuming active design meeting the need for comfort. This has resulted in the new paradigm of intelligent energy use. Communities and dwellings should employ new methods such as sensors, storage batteries and energy converters to improve the home environment [1]. This study advocates the development of a “bio-hydrogen energy based autonomous house” employing alternative energy in conjunction with environmental sensors, computing technology and active architectural elements to improve on some of the performance shortcomings of passive houses.

### 2. Literature Retrospective

An “autonomous house” is defined as a house that can function independently of support and services from public facilities [2]. The autonomous house movement does not require users to lead solitary, penny-pinching lives, however. The key characteristic of an autonomous house is the use of green energy technology to reduce environmental load, while also creating a sustainable, high-quality, comfortable living environment. In the field of architecture, “autonomy” has two implications: autonomous control and self-sufficiency [3]. Autonomy means that one can independently manage one’s own affairs and make independent decisions without influence or control by others [4,5]. Self-sufficiency means that one can maintain self-sufficiency in resources such as food, water and energy [6].

Although autonomy and self-sufficiency are applied to different situations in different fields of study and subjects, they are in fact overlapping concepts that are somewhat hard to distinguish. In sociology, self-sufficiency is used to describe the lifestyle of people living on the margins of society [7]. In the area of welfare policies, self-sufficiency programs are intended to help low-income families to achieve economic independence [8,9]. In the area of urban planning, one proposal calls for the construction self-sufficient individual homes around a large common house containing shared facilities in order to overcome the alienation of modem subdivisions and create a cooperative living environment [10]. In architecture, autonomous lightweight houses refer to the dwellings of nomadic peoples. Of course nomadism is also seen as an exemplary self-sufficient lifestyle. In the field of environmental protection, autonomy has recently become a key principle in the green energy technologies and the application of water resources [11–14]. In The Netherlands, self-sufficiency is a conceptual framework including both technology and environmental policies [15]. The use of clean energy and household appliances are necessary conditions for a comfortable life [16]. Nevertheless, living in an autonomous house does not imply that the occupants must lead the lives of nomads or persons on the margins of society. Instead, an autonomous house applies alternative energy and other relevant technologies in line with the principle of autonomy and thereby reduces dependence on fossil fuels and lessens carbon dioxide emissions in order to ameliorate global warming, while still providing a high-quality living environment.

Many controversies still surround the pursuit of energy and resource autonomy. According to the 2004 book “*Why Globalization Works*” by the economist Wolf [17], an advocate of the market economy, the atomization of the global economy in to self-sufficient regions or individuals will cause the reversal and collapse of the globalization that is taken place since the 1960s, which will cause civilization to atrophy. China’s recent adoption of a protective food self-sufficiency policy intended to ensure food security was a strong blow against the market economy. Nevertheless, the single-minded promotion of a market economy is also a highly suspect strategy. In this time of international food shortages, Japan, which was originally self-sufficient in rice, has actually been using rice to produce auto fuel and has developed a “rice alcohol car.” But while this has broadened the applications and increased the value of food crops, skeptical Japanese researchers feel that this will increase food prices and result in difficult-to-resolve raw material shortages [18]. Thailand’s King Bhumibol advocates economic self-sufficiency emphasizing the regional or individual pursuit of energy and resource autonomy. Apart from the advantages of self-sufficiency and independent control, autonomy can also achieve energy conservation and reduced carbon emissions by limiting the trading and transport of energy and resources [19]. The field of economics is based on the premise that human wants are boundless yet resources are limited; it emphasizes the functioning of the market mechanism, but neglects the influence of such non-market factors as renewable resources and effect on the ecology on economic systems. The natural world ultimately supports human economic systems. The human exploitation of the natural environment during the last forty or fifty years has caused great destruction and is very likely to have irreversible consequences [20]. Such matters as autonomy, whether to pursue localization or globalization and whether to favor economic self-sufficiency or a market economy remain highly controversial. Finally, how to employ architectural design to maintain autonomy in energy and resources is a very challenging issue.

The term “autonomous house” was first proposed by Alexander Pike, whose research goal was to develop a house service system that could reduce the consumption of local resources [21]. Vale in 1975 defined an autonomous house as a house that could function independently and required no inputs from nearby public service facilities. This type of house did not need to be connected with such services as gas, water, power, or sewers; it used alternative energy, such as solar power or wind power and could treat its own wastewater and sewage. It therefore produced no pollution and did not waste any energy. The first autonomous house consistent with theory was designed and built in 1993 by the autonomous house originators Brenda and Robert Vale [22]. This house achieves autonomy in the areas of water, energy and sewage and wastewater treatment, while also producing power for the use of the city. Of course many built structures consistent with the principle of autonomy have long existed in natural ecological systems. For instance, termite mounds employ some of the key principles of passive design. Table 1 presents four structures embodying the principles of autonomous design and discusses their function/ size, location, key technologies, design principles and research significance.

## 3. Theoretical Framework

**Figure 1.**
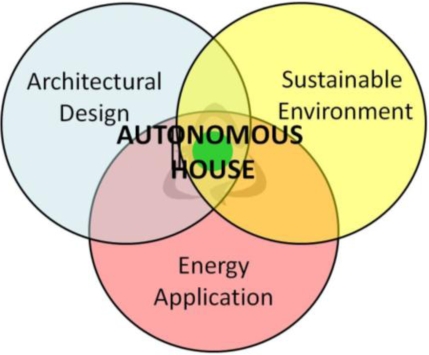
Macroscopic perspective of autonomous houses.

**Figure 2.**
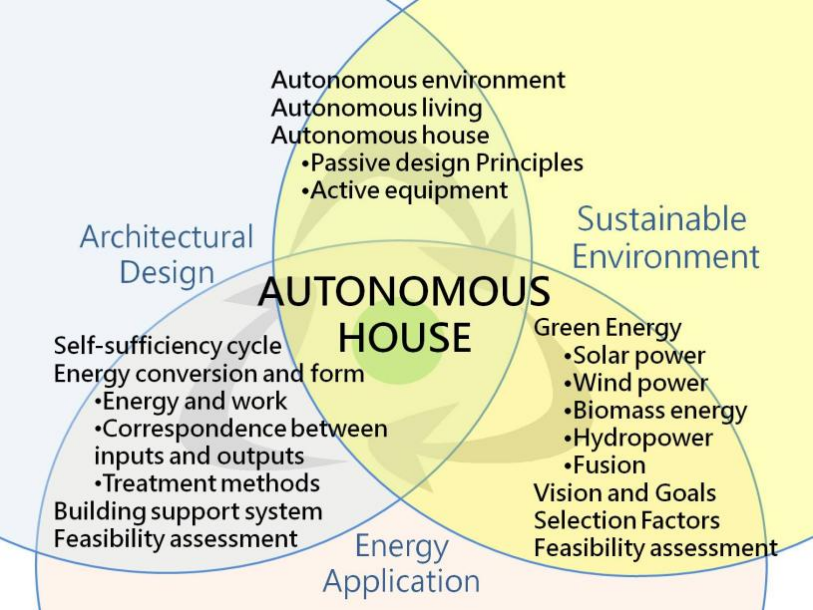
Micro items in autonomous houses.

In accordance with the definition of autonomous house, a retrospective of the literature and case analysis, from a macroscopic viewpoint, autonomous house design involves the three areas of sustainable environment, architectural design and energy applications (Figure 1). Turning to a micro viewpoint, sustainability and energy applications considerations include (1) green energy technology (renewal energy sources such as solar power, wind power, biomass energy, hydropower (including potential difference and tidal power) and fusion), (2) vision and goals, (3) green energy selection factors and feasibility assessment. With regard to energy applications and architectural design, items include (1) self-sufficiency cycle, (2) energy conversion and form (including energy and work conversion and calculation, correspondence between inputs and outputs and treatment methods), (3) building support system and (4) feasibility assessment. Architectural design and sustainability considerations include (1) autonomous environment (house location, layout and size), (2) autonomous living (independent dwellings or cooperative community form) and (3) autonomous house (passive design principles, supplementary use of active equipment to boost performance) (Figure 2).

## 4. Simulation and Empirical Research

### 4.1. Vision and Goals

The bio-hydrogen energy based autonomous house assumes that within ten years hydrogen energy technology will have reached a level of maturity allowing use in many everyday life applications. When that time comes, every family will be able to install a “hydrogen-generating fermentation tank” similar to a septic tank and hydrogen fuel cells about the size of window-mounted air conditioners. If the design goal of hydrogen power generation meeting the average household power need of 3 kW can be achieved, one fewer thermal power plant (i.e. generating an average of 600 MW) will be needed for every 200,000 households. Household-based distributed electrical systems can reduce dependence on large central power plants and thereby achieve the goals of energy conservation, reduced carbon emissions and energy autonomy.

### 4.2. Green Energy Technology and Bio-Hydrogen Energy

In view of the desire for autonomous household power generation and consumption, what form of alternative energy is most suitable as the primary household energy source? This question has long challenged energy specialists and economists. According to the recommendation of Feng Chia University’s bio-hydrogen energy application research team, the system with the greatest commercial potential is a “bio-hydrogen real-time power generation system” consisting of a dark fermentation hydrogen generation unit (continuously stirred anaerobic bioreactor, CSABR) and proton exchange membrane fuel cells (PEMFC). This type of system can generate renewable energy and also process urban waste and sewage. Green energy assessment factors include such objective conditions as the availability of raw materials, climate factors, site restrictions, production technology thresholds and unit costs. In comparison, although solar and wind power are easy to obtain, they are highly affected by the climate and relatively undependable. Hydropower is subject to site restrictions and nuclear energy has a far higher technological threshold than ordinary households can manage. From the point of view of household energy autonomy, biomass hydrogen production and power generation offers the advantages of easily acquired raw materials, freedom from climatic influences, stable power output, lack of site restrictions and a relatively low production technology threshold.

This study recommends the use of biomass energy systems involving the conversion of biomass to hydrogen, which is then stored in high density form and ultimately converted to forms of energy that can be used by the household. Suitable types of biomass include animal manure, crop waste, wood, sugar crops, urban garbage, urban sewage, aquatic plants and energy crops. Of these, urban waste such as urban garbage and urban sewage will play the most important role. It is estimated that Taiwan currently produces six million tons of organic waste each year that could be used as the raw material for biomass energy production.

### 4.3. Self-Sufficiency Cycle

**Figure 3.**
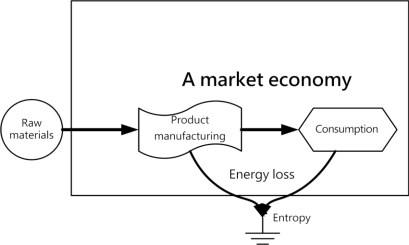
A market economy.

**Figure 4.**
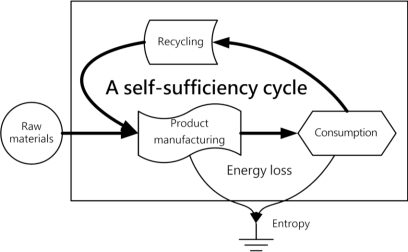
A self-sufficiency cycle.

In order to comply with the principles of energy self-sufficiency, a bio-hydrogen based autonomous house should complete a self-sufficiency cycle encompassing energy production, storage, distribution control, load applications, recycling, disposal and reuse. A life-support environment refers to an ecosystem in the earth’s biosphere that can meet the physiological needs of living organisms. Economic systems must obtain life-support functions from the natural environment and otherwise cannot survive. The basic theme of the sustainable development concept is that the effects of human actions should be subject to certain constraints so as not to destroy the diversity, complexity and functions of ecological life-support systems [26]. As a consequence, an effectively-functioning autonomous system based on a human society must integrate ecological and economic aspects if it is to realize the ideals of sustainable development. Figure 3 shows that the world’s resources and energy will be quickly exhausted due to the effect of entropy in a market economy emphasizing only the process encompassing only raw materials, product manufacturing and consumption [27]. Figure 4 shows how sustainable development must integrate ecology and economics, emphasizes recycling and reuse after consumption and relies on a self-sufficiency cycle to reduce energy consumption and slow entropy.

### 4.4. Building Support System

**Figure 5.**
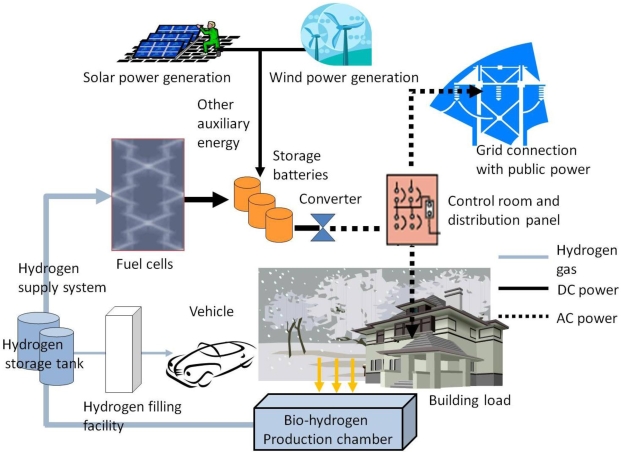
Building Support System.

**Figure 6.**
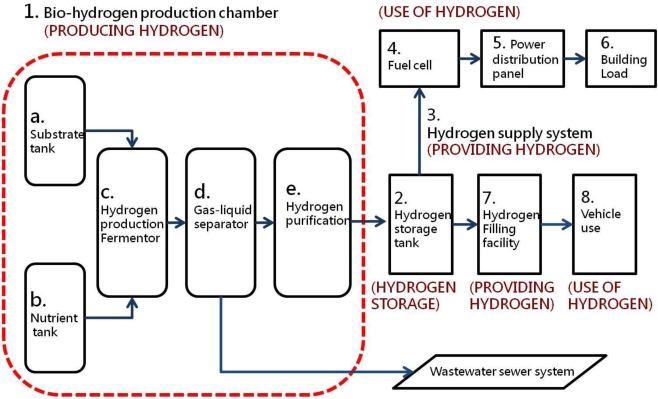
Bio-hydrogen real-time power generation system.

In accordance with the energy self-sufficiency cycle, a building can be seen as a means of converting mass to energy and must a cycle consisting of energy production, storage, distribution control, load applications, recycling, disposal and reuse. A building should also possess a support system including: (1) a bio-hydrogen production chamber, (2) a hydrogen storage tank, (3) a hydrogen supply facility, (4) fuel cells, (5) other auxiliary alternative energy sources (solar power, wind power, etc.), (6) storage batteries, (7) a converter, (8) a control room and description panel and (9) the building’s power load. If hydrogen fuel cell vehicles are used in the future, then (10) a hydrogen filling facility can be added. If the amount of power generated by the building may exceed its consumption and can be provided to other local users, then a grid connection to the public power supply can be added (Figure 5). The biology-hydrogen production chamber consists of five main components: (a) substrate tank, (b) nutrient salt tank, (c) hydrogen production fermentation tank, (d) gas-liquid separation tank and (e) a hydrogen purification device. The bio-hydrogen real-time power generation system includes hydrogen production, hydrogen storage, hydrogen supply and hydrogen use processes (Figure 6).

### 4.5. Feasibility Assessment

This section seeks to derive on the basis of the design goal of bio-hydrogen power generation meeting the average household power need of 3 kW what support equipment will be needed, the volumes of facilities, areas and layout and a plan diagram of the building. This information will provide a reference for the design of single-family autonomous houses.

**Figure 7.**
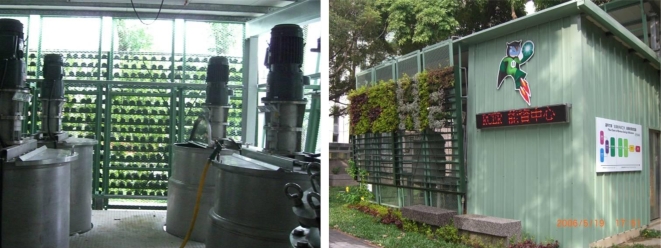
Experimental bio-hydrogen production plant (Left, Interior; Right, Exterior).

According to information from the Taiwan Power Co. [28], statistics for the most recent five years indicate that a household needs 3–4 kW of installed capacity. According to Lin [29], a 3.2 m^3^ hydrogen production fermentation tank can meet the power needs of an ordinary family. If, however, biomass is selected as a raw material, variables such as processing and conversion methods and environmental factors (temperature, humidity and pressure, etc.) will affect the rate of hydrogen production and hydrogen density. The installation of a hydrogen storage tank can resolve the problems associated with variable production rate. Excess hydrogen can be stored and used at times when the amount generated is insufficient. According to the bio-hydrogen real-time power generation system, developed by Feng-Chia University, during 300 days of use, each liter of the bio-hydrogen production tank generated 1.15±0.08 liters of hydrogen per hour. When small LEDs were hooked up to the system operating at ambient temperature (25°C), the current and voltage were 0.38 A and 2.28 V respectively. According to the formula power = current × voltage, the system generated an average of 0.87 W (0.38 × 2.28 = 0.87 W). It can therefore be conservatively estimated that a hydrogen production fermentation tank with a volume of 3,222 L (≈3.2 m^3^) will be needed to accommodate an average household load of 3 kW ((3000/0.87) ÷ (1.15–0.08) = 3,222 L ≈ 3.2 m^3^). This is roughly the size of an ordinary commercial septic tank (2–3 m^3^). Taking Feng Chia University’s experimental bio-hydrogen production plant as an example (Figure 7), the five main components of the bio-hydrogen production chamber (substrate tank, nutrient salt tank, hydrogen production fermentation tank, gas-liquid separation tank and hydrogen purification device) have volume ratios of 2:2:1:1:1. As a consequence, the bio-hydrogen production chamber will have a total volume seven times that of the hydrogen production fermentation tank, i.e. 22.4 m^3^. Assuming an ordinary house with vertical clearance of 2.5 m, approximately 15 m^2^ of equipment area will be needed (assuming that the tank is 1.5 m in height). If passageways and other equipment occupy one-fourth of the bio-hydrogen production room, then the room will require a total usable area of 20 m^2^). Commercial 3 kW fuel cells have a volume of approximately 0.33 m^3^ (http://www.solore.com.tw/power/fuel/stacks/3kw.htm). Hydrogen storage tanks should be able to store enough hydrogen for three days. Since an average household in Taiwan uses roughly 3 × 320 ÷ 30 = 32 kWh every three days and a 3 kW fuel cell requires 36 L of hydrogen per minute, 36 L of hydrogen can therefore generate 0.05 kWh. Approximately 32 ÷ 0.05 = 640 L of hydrogen will therefore be needed for three days. A commercial hydrogen storage tank with a volume of roughly 1.68 m^3^ (http://www.hbank.com.tw/fc_products_pr_05.htm) can therefore be used in this application. According to actual data from the FCU Research Center for Energy & Resources, when the working volume is 3 l and the HRT is 8 h, the feed matrix concentration will be 20 g COD/L, and the system will generate 0.87 W of electrical power. Furthermore, since 20 g COD/L = 17.8 g sucrose/L (actual data), 6.675 g sucrose/h (3–1/8 h x 17.8 g sucrose/L = 6.675 g sucrose/h) will be needed to generate 0.87 W, and 23,017 g sucrose/h will be needed to generate 3 kW (6.675 g sucrose/h x 3000/0.87 = 23,017 g sucrose/h). According to the Taiwan Power Corp., average daily household power consumption is 10 kWh, so the system must run for 3.3 h daily to meet a daily household electrical load of 3 kW (10 kWh/3 kW). A total of 75,956 g of sucrose will therefore be needed per household per day (23,017 g sucrose/h × 3.3 h/day = 75,956 g sucrose/day).

**Figure 8.**
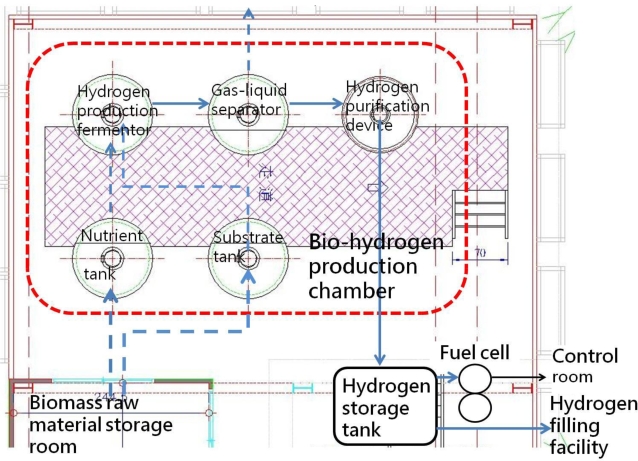
Schematic plan of a bio-hydrogen real-time power generation system.

Table 2 gives the sizes of volumes and areas of a biomass raw material storage room, bio-hydrogen production room, fuel cells, hydrogen storage tank and control room, along with a model. Figure 8 shows a schematic plan of a bio-hydrogen real-time power generation system as a reference for the design of single-family autonomous houses.

### 4.6. Autonomous Control

In accordance with the principles of energy autonomy, a house design geared towards user needs, must, in addition to complying with passive building layout and design principles, also consider the use of active adaptive devices. Active devices can be used to boost the performance of a passive building, enhance the autonomous control of energy applications and maintain a comfortable living environment.

**Figure 9.**
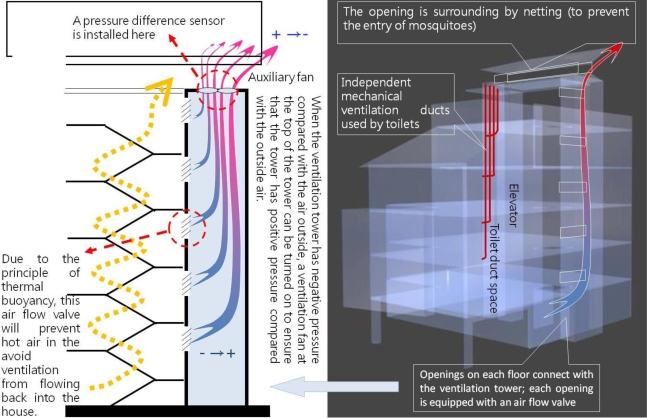
Design of thermal buoyancy ventilation tower (drawn by Chen Nien-Tzu).

The autonomous house in this project will employ thermal buoyancy ventilation using the stairway as a ventilation tower. Due to the effect thermal buoyancy, hot air will normally enter the ventilation tower via the stairway and will escape from the top of the tower due to the effect of air flow. When external pressure is greater than indoor pressure, however, reverse air flow may occur in thermal buoyancy ventilation and hot air will be unable to escape. When pressure difference sensors and computing technology are used in conjunction with an air flow valve, if the ventilation tower has negative pressure compared with the air outside, a ventilation fan at the top of the tower can be turned on, or the angle of the air flow valve adjusted, to insure that the interior of the tower has positive pressure compared with the outside air and the hot air will be able to easily escape. Because of this, the autonomous house will use an active device to ensure that the passive thermal buoyancy ventilation tower achieves optimal ventilation performance (Figure 9) [30].

## 5. Recommendations and Conclusions

The study establishes the outline of a feasible hydrogen energy-based autonomous house that will produce no pollution and waste no energy. Suggestions for future research are as follows:

(1) *Independent island-type residential power generation and use model:*

This study has focused on the development of autonomous urban residential linked to public power system sand it is hoped that household-based distributed electrical systems can reduce dependence on large central power plants. Houses located in remote suburbs and places where public power is not easily available have an even greater need for autonomous energy systems, however. Nevertheless, further research must study how to maintain the stability and performance of energy output, direct use, storage and supply for use. Research may also focus on the modification of household generating systems to supply AC power and examine the use and distribution models, along with household appliances and equipment, which are suitable for AC power.

(2) *Integration and management of multiple energy systems:*

In accordance with the principles of energy autonomy, buildings can support multiple energy sources (such as biomass energy, solar power, wind power, hydropower and geothermal power, etc.). Further research should therefore focus on the stable use of multiple energy sources and different types of current that they produce (AC or DC). More efficient energy management platforms will be needed to avoid unnecessary losses during energy conversion.

(3) *Examining the ecological characteristics of urban systems from the point of view of energy:*

According to the broad definition of an ecosystem, cities can be considered a part of an ecosystem. Further research can examine the metabolism of matter, conversion of energy, cycling of water and flow of currency in urban production and consumption activities and investigate the dynamic mechanisms, functional principles and economic and ecological benefits, spatial structures and control rules of urban systems.

(4) *Handling of wastewater and chemical oxygen demand (COD) when biomass is used to produce hydrogen:*

Because Feng Chia University’s biomass hydrogen generator produces only a small amount of wastewater, this wastewater is mixed with campus domestic sewage and discharged directly to campus wastewater sewers; it is sent to the university’s wastewater treatment pond and then discharged into the city’s wastewater sewer system. It will be necessary to establish community-level wastewater treatment facilities, however, when biomass hydrogen production plants become common in the future. Such facilities must reduce the COD of wastewater from hydrogen production enough to meet emission standards, before the water can be discharged to city wastewater systems. Collaboration with environmental protection engineers will be needed in order to integrate relevant pollution control measures.

This research considers the bio-hydrogen energy based autonomous house to be a key next-generation dwelling technology. This has two implications: First, the house’s self-sufficient energy cycle consisting of production, consumption and recycling fulfills the needs of sustainable development. Secondly, the use of sensors, computing mechanisms and adaptive architectural elements will enable autonomous control of the environment. With regard to the application and reuse of energy and resources, an autonomous house of this type can harmonize a passive energy conservation design with the energy demands of active devices meeting the need for a comfortable environment.

## Figures and Tables

**Table 1.** Analysis of buildings applying autonomous principles.

Name Item	Mounds [23]	The Autonomous House [22]	Hockerton Housing Project [24]	Self-Sufficient Skyscraper [25]
Illustration	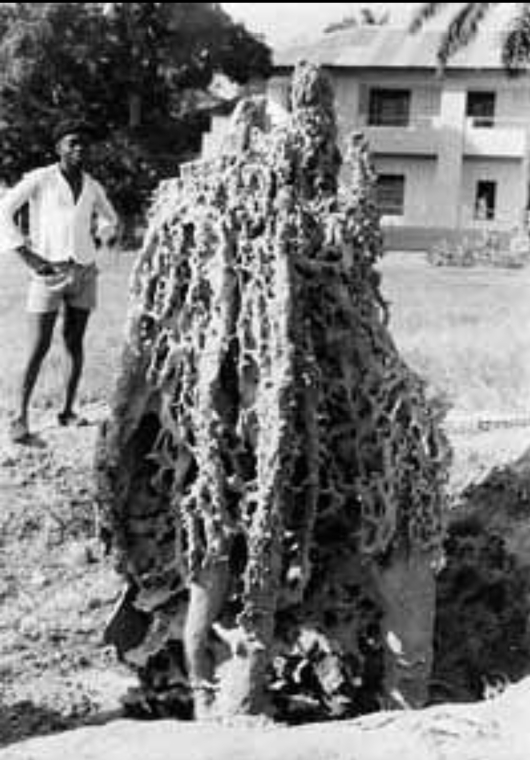	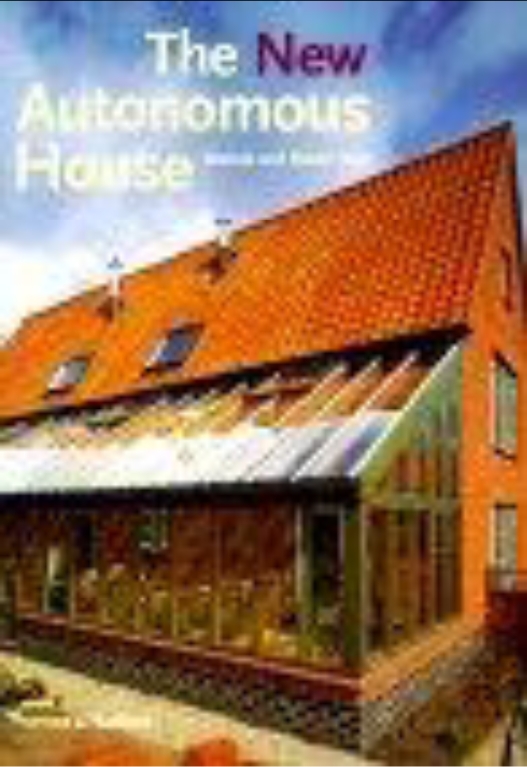	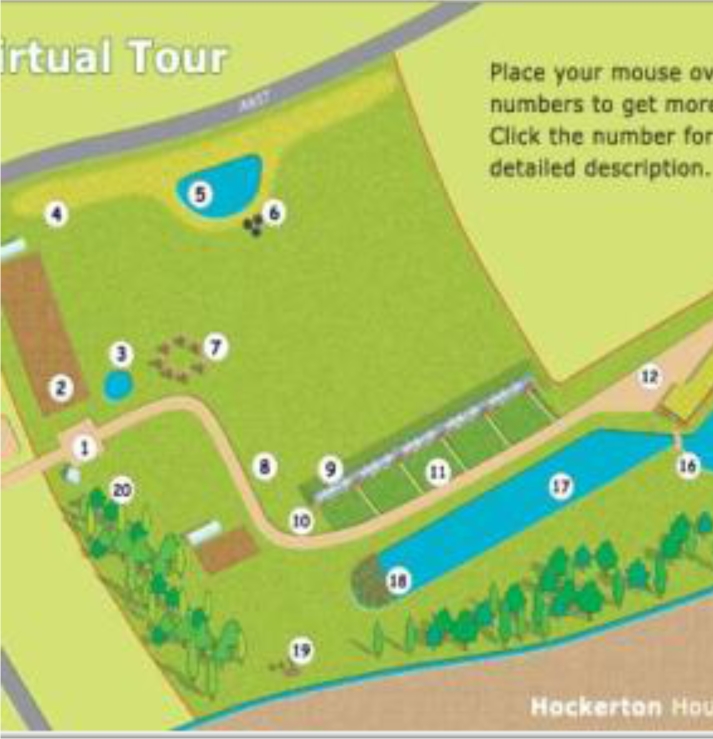	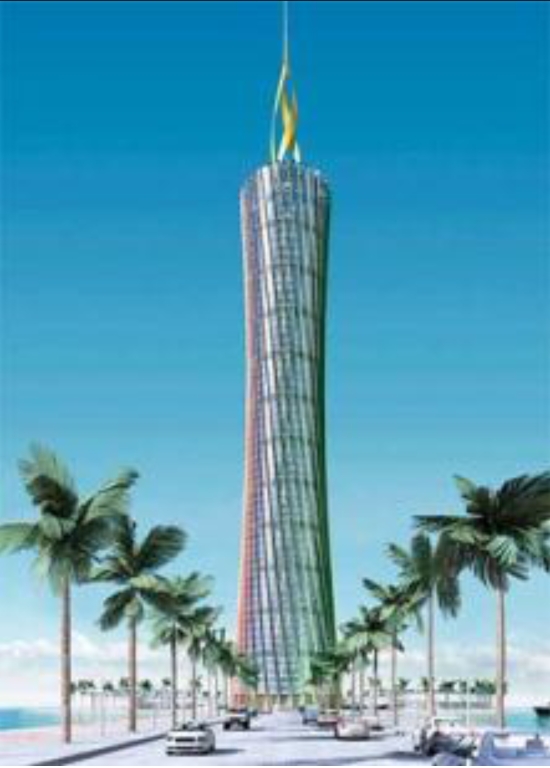
Designer/year	Termites/unknown	Brenda and Robert Vale/1993	Brenda and Robert Vale/ 1998	Matthew Sparkes/planned
Function	Underground home	Private home	Rental community	Office tower
Location	Africa	Nottinghamshire, England (city center)	Hockerton, England (suburbs)	Riyadh, Dubai and Bahrain (seaside)
Key technologies and design principles	Termite mounds have a passive design that regulates air flow and conserves energy. The mounds give the termites autonomy: Apart from providing a comfortable living environment, the mounds also facilitate the growth of fungi (which dispose of the termites’ wastes).	Energy is obtained from the sun and wind; rainwater is collected for use as drinking water. The house is built using recycled and local materials as much as possible.	The community’s energy and water supplies and wastewater treatment, are supplied by a zero carbon dioxide system; food is grown using permaculture technology. The community consists of five modular single-story backfilled. The modular design makes the homes easier to build and cuts costs.	The cylindrical shape of the tower exposes the minimum surface area to the sun and thereby reduces air conditioning energy needs. The roof has a wind turbine and solar panels and storage batteries for emergency use. Solar panels on the sea provide energy from hydrogen extracted from seawater. Energy is stored in hydrogen fuel cells for nighttime use.
Research significance	Scans and computer simulations of termite mounds have provided a research model for passive energy conservation and waste disposal.	Located in the middle of a modern Western city, this house demonstrates an autonomous and sustainable lifestyle.	House construction, community planning and lease contract restrictions shape this cooperative, autonomous community.	Employs modern green technology, supports sustainable environment development and creates a high-quality, comfortable living environment.

**Table 2.** Estimates of functional areas and the study model.

Function	Volume	Area	
Biomass raw material storage room	7.5 M^3^	3 M^2^	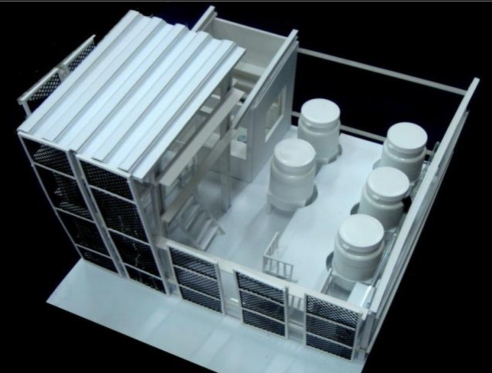
Bio-hydrogen production chamber	22.4 M^3^	20 M^2^	
Fuel cells	0.33 M^3^	0.5 M^2^	
Hydrogen storage tank	1.68 M^3^	1.5 M^2^	
Control room	7.5 M^3^	3 M^2^	

## References

[1] Capelli L, Guallart V, Iaac (2006). Self-Sufficient Housing.

[2] Vale B, Vale R (1975). The Autonomous House: Design and planning for self-sufficiency.

[3] Harper P (2002). Sufficient Unto Itself the New Autonomous House.

[4] *The American Heritage* Available online: http://en.wikipedia.org/wiki/Self-sufficiency (accessed February 2009).

[5] Yourictionary.com. Available online: http://www.yourdictionary.com/ahd/s/s0244800.html (accessed December 2008).

[6] Moench M (2004). Self-Sufficient Homes. Futurist.

[7] Smith DP (2007). The ‘buoyancy’ of ‘other’ geographies of gentrification: Going ‘back-to-the water’ and the commodification of marginality. Tijdschrift Voor Economische En Sociale Geografie.

[8] Anthony J (2005). Family self-sufficiency programs - An evaluation of program benefits and factors affecting participants’ success. Urban Aff. Rev.

[9] Lindbergh L, Larsson CG, Wilson TL (2004). Cost control and revenue generation: The case of public-housing companies’ experiences in Sweden. Reg. Stud.

[10] Stewart D (2002). Habitat and ecology: The co-housing experiment in the United States. Rev. Fran. Etud. Amer.

[11] Lampinen A (2004). Biogas farming: An energy self-sufficient farm in Finland. Refocus.

[12] Sartori I, Hestnes AG (2007). Energy use in the life cycle of conventional and low-energy buildings: A review article. Energ. Bldg.

[13] Ulleberg O, Morner SO (1997). TRNSYS simulation models for solar-hydrogen systems. Solar Energ.

[14] Voss K, Goetzberger A, Bopp G, Haberle A, Heinzel A, Lehmberg H (1996). The self-sufficient solar house in Freiburg - Results of 3 years of operation. Solar Energ.

[15] Melchert L (2007). The Dutch sustainable building policy: A model for developing countries. Bldg. Environ.

[16] Chen SY *The study of applying agent-based theory to adaptive architectural environments—Smart skin as an example*; Ph.D. thesis, National Cheng Kung University: Taipei, Taiwan, 2007 2 4

[17] Wolf M (2004). Why Globalization Works.

[18] *Japan’s Extravagant Experimental Use of Rice Wine to Make Gasoline Attracts Widespread–Controversy International Headline Report* Available online: http://www.gog.com.cn (accessed July 2008).

[19] *Sung Tung, King Bhumibol of Thailand: Self-sufficiency Can Prevent Stumbles* Available online: http://www.cw.com.tw/article/print.jsp?id=34792 (accessed 2008).

[20] Huang SL (2004). Energy Basis for Urban Ecological Economic System.

[21] Pike A (1972). Friend of the earth.

[22] Brenda RV (2000). The New Autonomous House.

[23] *Termites Could Hold the Key to Self-Sufficient Buildings.* Available online: http://www.epsrc.ac.uk/PressReleases/TermitesCouldHoldTheKeyToSelfSufficientBuildings.htm (accessed January 2009).

[24] *Hockerton Housing Project* Available online: http://www.hockertonhousingproject.org.uk/ (accessed December 2008).

[25] Sparkes M *Self-Sufficient Skyscraper.* Available online: http://www.treehugger.com/files/2007/05/selfsufficient.php (accessed December 2008).

[26] Costanza R (1991). Ecological Economics: The Science and Management of Sustainability.

[27] Odem HT (1983). System Ecology.

[28] Taiwan Power Co Taiwan’s Performance during the Most Recent Five YearsAvailable online: http://www.taipower.com.tw/left_bar/jing_ying_ji_xiao/5year_effects.htm (accessed January 2009).

[29] Lin CN, Wu SY, Lee KS, Lin PJ, Lin CY, Chang JS (2007). Integration of fermentative hydrogen process and fuel cell for on-line electricity generation. Int. J. Hydrogen Energ.

[30] Chiang CM, Chen CH, Hsieh Y, Kao CY, Chen NT, Chen SY, Wang WJ The Case Study of Sustainable Healthy Housing Fitting in with Subtropical Taiwan’s Climate and Geography.

